# ﻿The tropiduchid planthopper genus *Connelicita* Wang & Bourgoin, 2015: two new species from Central Vietnam and new records (Hemiptera, Fulgoromorpha, Tropiduchidae)

**DOI:** 10.3897/zookeys.1186.113701

**Published:** 2023-12-12

**Authors:** Jérôme Constant, Thai-Hong Pham, Cuong Viet Canh Le, Trung Thanh Vu, Hoai Thu Thi Nguyen

**Affiliations:** 1 Royal Belgian Institute of Natural Sciences, O.D. Taxonomy & Phylogeny – Entomology, Vautier street 29, B-1000 Brussels, Belgium Royal Belgian Institute of Natural Sciences Brussels Belgium; 2 Mientrung Institute for Scientific Research, Vietnam National Museum of Nature, VAST, 321 Huynh Thuc Khang, Hue, Vietnam Mientrung Institute for Scientific Research, Vietnam National Museum of Nature Hue Vietnam; 3 Graduate School of Science and Technology, Vietnam Academy of Science and Technology, 18 Hoang Quoc Viet, Hanoi, Vietnam Vietnam Academy of Science and Technology Hanoi Vietnam; 4 Vietnam National Museum of Nature, Vietnam Academy of Science and Technology (VAST), 18 Hoang Quoc Viet, Hanoi, Vietnam Vietnam National Museum of Nature, Vietnam Academy of Science and Technology Hanoi Vietnam

**Keywords:** Bach Ma National Park, biodiversity, Fulgoroidea, Indochina, Phong Dien District

## Abstract

Two new planthopper species of the tropiduchid genus *Connelicita* Wang & Bourgoin, 2015, *C.bachmaensis* Constant & Pham, **sp. nov.**, and *C.phongdienensis* Constant & Pham, **sp. nov.** are described from Bach Ma National Park and from the Centre for Conservation of Vietnam Natural Resources and Rescue of Animals and plants, Phong Dien District in Thua Thien-Hue Province, respectively. These new records greatly extend the distribution of the genus, which was known from southern China (Guangxi) and North Vietnam, to the south, reaching the mid area of Central Vietnam. New records are provided for *C.haiphongensis* Wang & Zhang, 2015, extending the distribution of the species from Cat Ba Island to a large zone in North Vietnam. Illustrations of habitus, details, and male genitalia are given as well as a distribution map and photographs of the habitat. An identification key to the species of Vietnam is provided. The genus *Connelicita* now comprises five species.

## ﻿Introduction

The family Tropiduchidae is distributed worldwide and contains 680 species in 197 genera, including 26 species in Vietnam (Bourgoin 2023). Within the subfamily Elicinae Melichar, 1915, the tribe Elicini Melichar, 1915 counts 143 species in 37 genera distributed in most zoogeographical regions except the Palaearctic (Wang et al. 2015; Bourgoin 2023).

The genus *Connelicita* Wang & Bourgoin, 2015 was rather recently described to accommodate three species: one from southern China (Guangxi) and two from North Vietnam (Wang et al. 2015).

Study of the recent material of Tropiduchidae in the collections of Vietnam National Museum of Nature and Royal Belgian Institute of Natural Sciences revealed two undescribed species of *Connelicita* from Central Vietnam and several additional records of *C.haiphongensis* Wang & Zhang, 2015.

The present paper aims to describe the two new species as a new contribution to the Vietnamese tropiduchid fauna.

## ﻿Materials and methods

The genitalia were extracted after soaking the abdomen for some hours in a 10% solution of potassium hydroxide (KOH) at room temperature. The pygofer was separated from the abdomen, thoroughly rinsed in 70% ethanol, and the aedeagus dissected with a needle blade for examination. The whole was then placed in glycerin for preservation in a tube attached to the pin of the corresponding specimen. Photographs of collection specimens were taken with a Leica EZ4W stereomicroscope, stacked with CombineZ, and optimized with Adobe Photoshop; photographs from the field were taken with an Olympus Tough 6 camera. The map was produced with SimpleMappr (Shorthouse 2010) and includes records available from Wang et al. (2015). The external morphological terminology follows O’Brien and Wilson (1985), the wing venation terminology follows Bourgoin et al. (2015), and for the male genitalia, Bourgoin and Huang (1990). The classification used follows FLOW (Fulgoromorpha Lists on The Web – Bourgoin 2023). The metatiobiotarsal formula gives the number of spines on (side of metatibia) apex of metatibia / apex of first metatarsomere / apex of second metatarsomere.

The measurements were taken as in Constant (2004) and the following acronyms are used:

**BB** maximum breadth of the body

**BF** maximum breadth of the frons

**BTg** maximum breadth of the tegmen

**BV** maximum breadth of the vertex

**LF** length of the frons at median line

**LT** total length (apex of head to apex of tegmina)

**LTg** length of the tegmen

**LV** length of the vertex at median line

Acronyms used for the collections:

**RBINS** Royal Belgian Institute of Natural Sciences, Brussels, Belgium

**VNMN** Vietnam National Museum of Nature, Hanoi, Vietnam

Other abbreviations

**CCRR** Centre for Conservation of Vietnam Natural Resources and Rescue of Animals and plants

## ﻿Taxonomy

### ﻿Family Tropiduchidae Stål, 1866

**Subfamily Elicinae Melichar, 1915**

**Tribe Elicini Melichar, 1915**

#### ﻿Genus Connelicita

Taxon classificationAnimaliaHemipteraTropiduchidae

Wang & Bourgoin, 2015

CCFC7C2B-24E6-5B6B-913B-748C270F549C

##### Type species.

*Connelicitabackyensis* Stroiński & Bourgoin, 2015 by original designation.

##### Diagnosis.

Head capsule with frons widely developed below level of eyes; anterodorsal part of genae visible in dorsal view. Tegmina with costal area containing more than 16 cells, cells longer than wide; ScP regularly straight and presence of 2 or 3 veinlets pcu-cup.

##### Distribution.

China: Southern China (Guangxi); Vietnam: North and Central.

##### Species included.

*Connelicitabachmaensis* Constant & Pham sp. nov.

*Connelicitabackyensis* Stroiński & Bourgoin, 2015

*Connelicitahaiphongensis* Wang & Zhang, 2015

*Connelicitalungchowensis* (Chou & Lu, 1977)

*Connelicitaphongdienensis* Constant & Pham sp. nov.

### ﻿Key to the species of *Connelicita* Wang & Bourgoin, 2015 from Vietnam

**Table:** 

1	Anal tube in lateral view strongly narrowing in distal half (*An* – Fig. 9A); dorsal process of periandrium spinose (*dpp* – Fig. 9E)	**2**
–	Anal tube of male subcylindrical and elongate (*An* – Fig. 3A–C); dorsal process of the periandrium forming a large lobe concave in distal portion (*dpp* – Fig. 3F, G)	***C.bachmaensis* Constant & Pham, sp. nov.**
2	Capitulum of the gonostylus with two spines and without digitiform process (Wang et al. 2015: figs 12, 31); spinose dorsal process of the periandrium moderately developed (Wang et al. 2015: figs 11, 30)	**3**
–	Capitulum of the gonostylus with one spine and a dorsal digitiform process (*ca* – Fig. 9A–D); spinose dorsal process of the periandrium strongly developed (*dpp* – Fig. 9E)	***C.phongdienensis* Constant & Pham, sp. nov.**
3	Capitulum of the gonostylus with two spines very unequal in size, proximal one much stronger than distal one (Wang et al. 2015: fig. 31); distal portion of the gonostylus in lateral view, rounded and developed dorsocaudad (Wang et al. 2015: fig. 31); in dorsal view, posterior and lateral margins of the anal tube concave in distal half (Wang et al. 2015: fig. 29)	***C.backyensis* Stroiński & Bourgoin, 2015**
–	Capitulum of the gonostylus with two spines more or less equal in size (Wang et al. 2015: fig. 12); distal portion of gonostylus in lateral view, rounded and developed caudad (Wang et al. 2015: fig. 12); in dorsal view, posterior and lateral margins of the anal tube rounded in distal half (Wang et al. 2015: fig. 10)	***C.haiphongensis* Wang & Zhang, 2015**

#### ﻿ Connelicita bachmaensis

Taxon classificationAnimaliaHemipteraTropiduchidae

Constant & Pham sp. nov.

05102608-B693-55EC-9C9B-FD00890D76A7

https://zoobank.org/32D5F308-98C3-465F-85FE-C96DC1237A87

**Figure 1.**
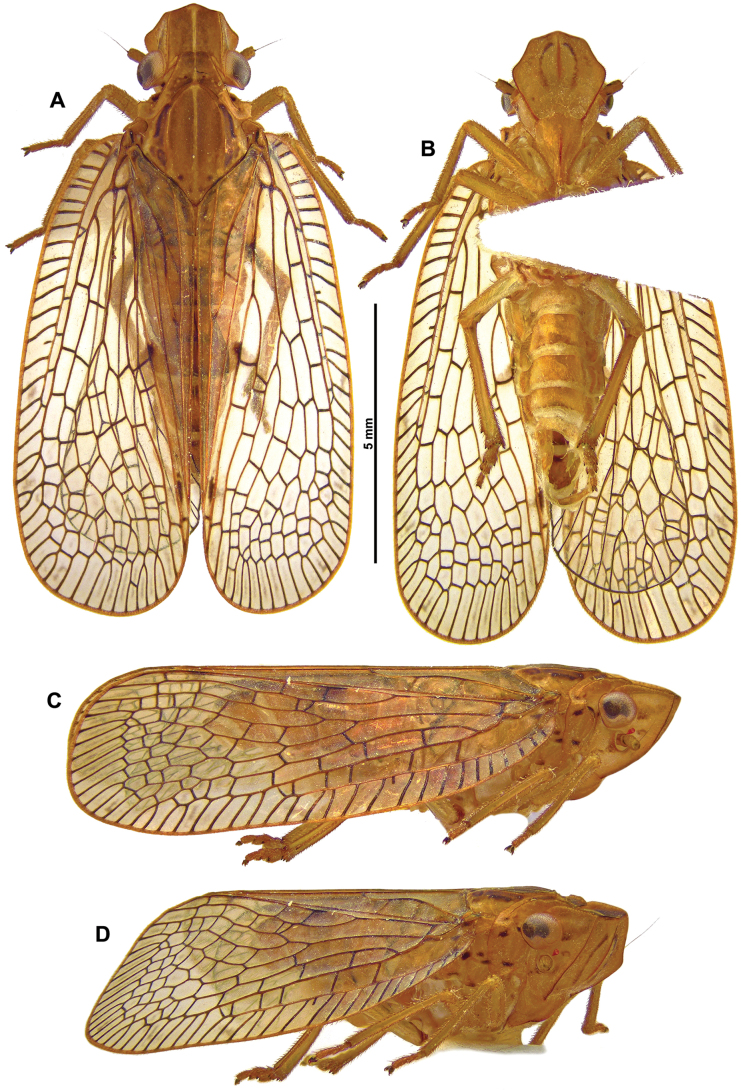
*Connelicitabachmaensis* sp. nov., holotype ♂ **A** habitus, dorsal view **B** habitus, ventral view **C** habitus, lateral view **D** habitus, anterolateral view.

**Figure 2.**
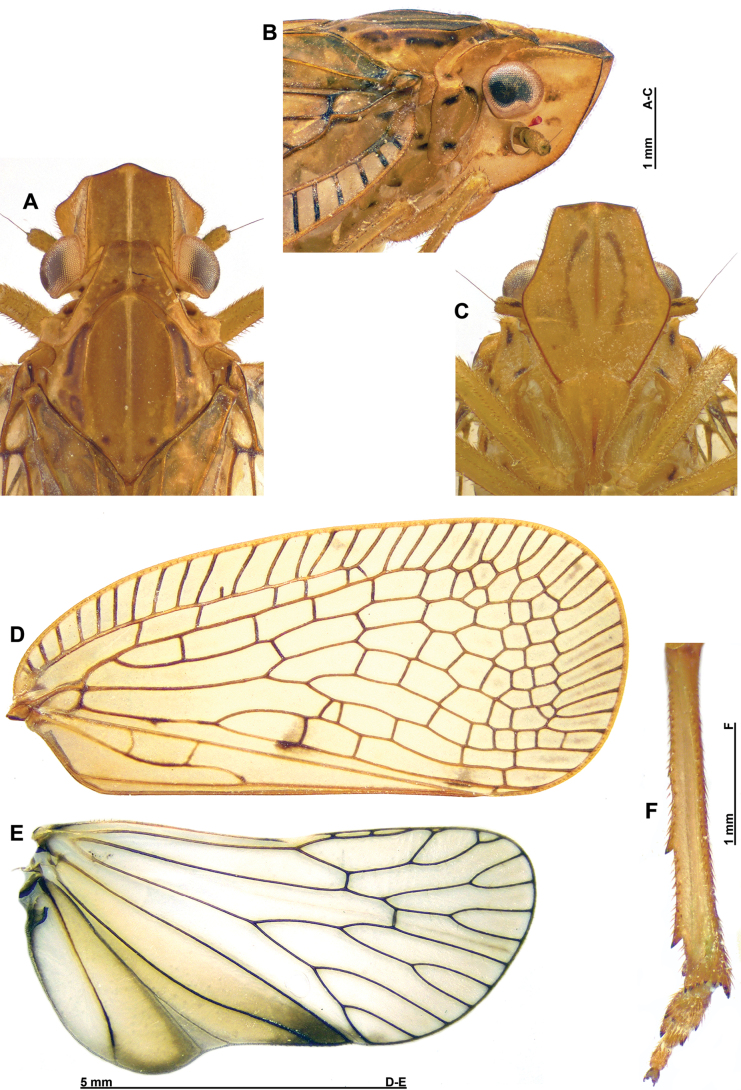
*Connelicitabachmaensis* sp. nov., holotype ♂ **A–C** detail of head and thorax **A** dorsal view **B** lateral view **C** perpendicular view of frons **D** right tegmen anterolateral view **E** right hind wing **F** right metatibia and metatarsus, ventral view.

**Figure 3.**
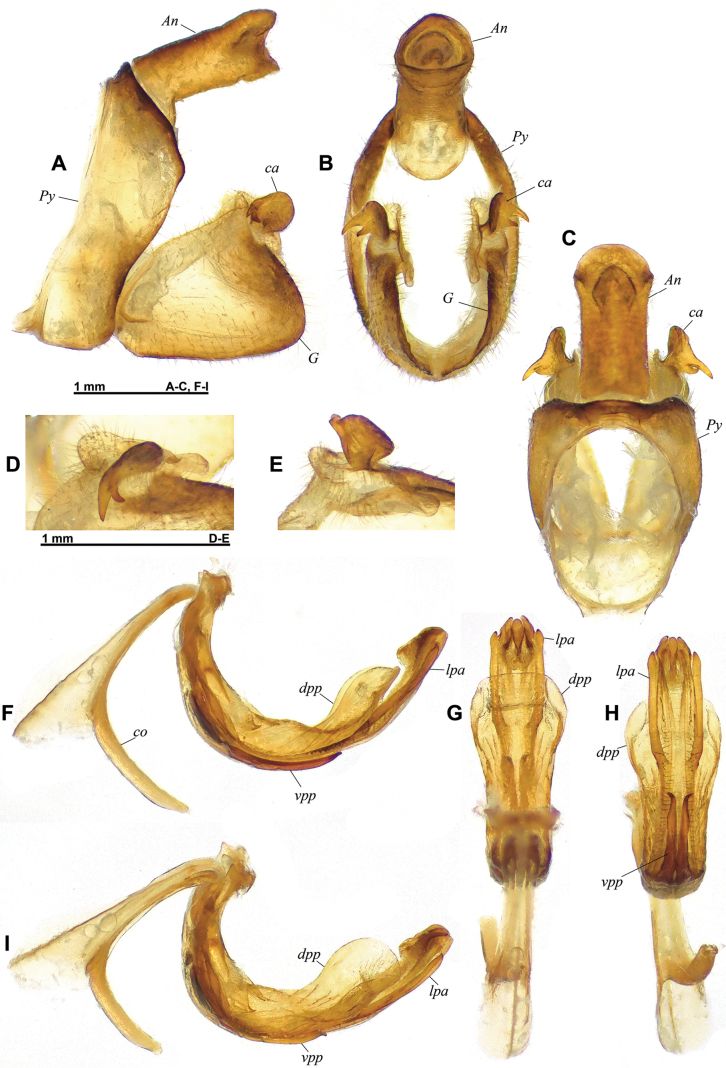
*Connelicitabachmaensis* sp. nov., holotype ♂, terminalia **A–C** pygofer, gonostyli and anal tube **A** lateral view **B** caudal view **C** dorsal view **D–E** capitulum of left gonostylus **D** left laterodorsal view **E** right laterodorsal view **F–I** aedeagus, phallobase and connective **F** left lateral view **G** anterodorsal view **H** posteroventral view dorsal view **I** left lateroventral view.

**Figure 4.**
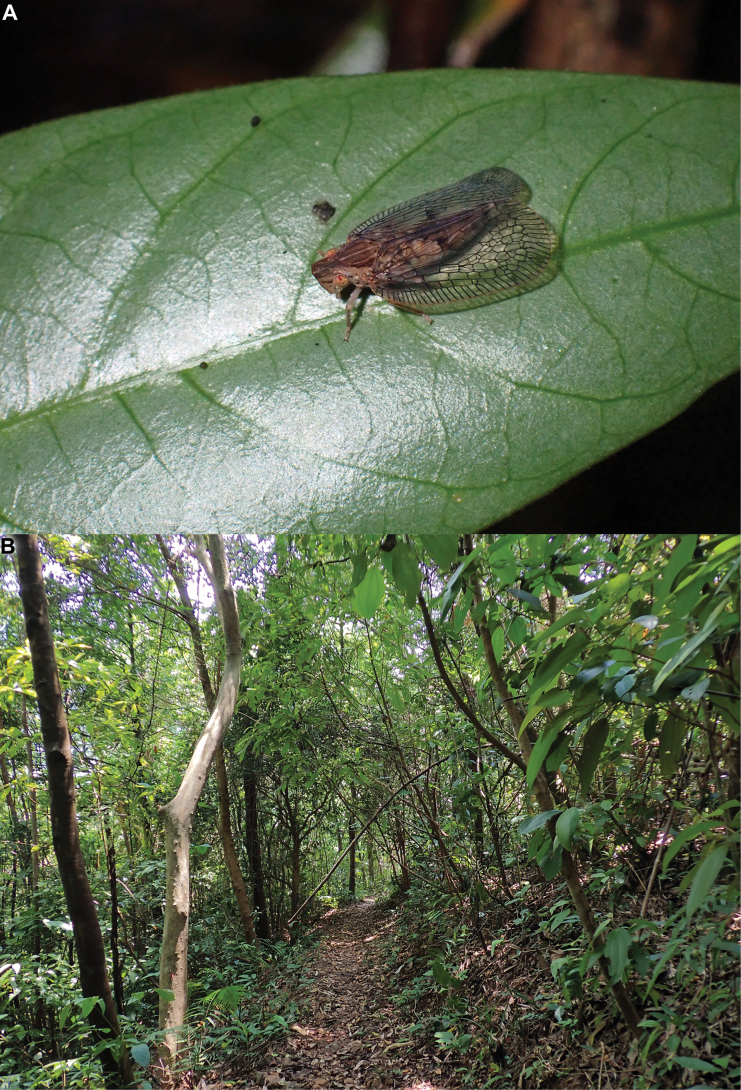
*Connelicitabachmaensis* sp. nov., Vietnam, Bach Ma National Park, Pheasant Trail, 12 May 2023 **A** adult specimen sitting on leave of unidentified plant **B** habitat.

**Figure 5.**
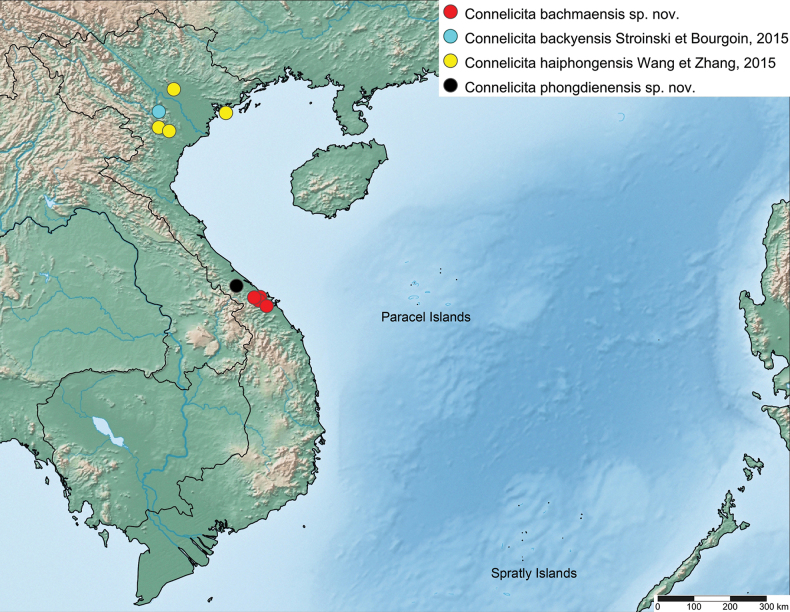
Distribution map of the species of *Connelicita* of Vietnam.

Figs 1 , 2 , 3 , 4 , 5

##### Type materials.

***Holotype*** ♂, Vietnam •– **Thừa Thiên-Huế Province** • Bach Ma National Park, Pheasant trail; 16°13'38"N, 107°51'20"E; 3 Mar. 2023; by net; Trung T. Vu leg.; VNMN.

***Paratypes***, Vietnam – **Thừa Thiên-Huế Province** • 3 ♀♀; same collection data as for holotype; VNMN • 2 ♂♂, 5 ♀♀; **Thừa Thiên-Huế Province** • Bach Ma National Park, Yes Hue Eco; 16°13'05"N, 107°42'27"E; 1 Jun. 2023; alt. 152 m; by net; Hoai T.T. Nguyen leg.; VNMN • 3 ♂♂, 1 ♀; Bach Ma National Park, Pheasant trail; 16°13'38"N, 107°51'20"E; 10–20 May 2023; alt. 500–600 m; J. Constant & L. Semeraro leg.; I.G.: 34.640; RBINS • 1 ♂, 1 ♀; Bach Ma National Park, near ranger station; 16°08'37"N, 107°49'36"E; 18 May 2023; alt. 300–600 m; J. Constant & L. Semeraro leg.; I.G.: 34.640; RBINS • 1 ♂; Bach Ma National Park, Yes Hue Eco; 16°13'05"N, 107°42'27"E; 17 May 2023; alt. 200–300 m; J. Constant & L. Semeraro leg.; I.G.: 34.640; RBINS – **Da Nang Province** • 1 ♂; Ba Na-Nui Chua; 16°00'N, 108°01'E; 16–19 Jul. 2017; GTI Project; J. Constant & J. Bresseel leg.; I.G.: 33.498; RBINS.

##### Diagnosis.

The species can be separated from all other species of *Connelicita* by the following features of the male terminalia: anal tube subcylindrical and elongate (~1.9× as long as wide in dorsal view), with dorsal margin not emarginate in lateral view (Fig. 3A–C), capitulum of gonostylus placed at apicodorsal angle and bearing two strong lateral teeth (Fig. 3A–C), and dorsal process of periandrium forming a large lobe concave in distal portion (Fig. 3F, G).

##### Description.

***Measurements and ratios***: LT: ♂ (*n* = 6): 12.56 mm (11.91–13.17); ♀ (*n* = 2): 13.60 (13.50–13.70). LTg/BTg = 2.19; LW/BW = 2.02; LV/BV = 1.04; LF/BF = 1.15.

***Head*** (Fig. 2A–C): narrower than thorax and elongate, with ~2/3 of vertex length surpassing eyes and genae largely visible from above. Vertex brown, with median yellowish line, weakly concave, ~1.0× as long in mid-line as broad basally, with lateral margins subparallel, anterior margin bisinuate, roundly produced anteriorly in middle portion, and posterior margin excavate. Frons pale yellowish brown, with two curved brown lines on disc; lateral margins finely lined in dark brown; convex in lateral view; smooth, with median carina reaching dorsal margin but not frontoclypeal suture. Genae yellowish, with brown markings between eye and anterior margin, between antennal insertion and posterior margin, and near red ocellus. Clypeus pale yellowish brown, triangular, with median carina in distal portion and with fronto-clypeal suture rounded. Labium yellowish, elongate, and narrow, reaching metacoxae, with apical segment elongate. Eyes globular (not emarginate) and protruding laterally; ocelli present. Antennae yellowish brown, with longitudinal black line along underside of pedicel; scape ring-shaped and pedicel cylindrical, longer than broad.

***Thorax*** (Fig. 2A, B): pronotum yellowish brown, darker on middle portion, with median, yellowish carina and 3 or 4 small yellowish tubercles on sides of disc; paranotal fields with 3 black spots, one being behind eye. Mesonotum yellowish brown, with median and peridiscal carinae yellowish; blackish, slightly curved longitudinal line in lateral fields and 2 blackish points at base of scutellum. Tegulae yellowish brown.

***Tegmina*** (Figs 1A–D, 2D): translucent, with brown spot near middle of vein CuP and vein CuA2 and apical cells weakly infuscate in middle; costal and postclaval margins slightly diverging towards the posterior; distal margin widely rounded.

***Venation*** (Fig. 2D): costal area well developed, with numerous cross-veins delimitating elongate cells, pterostigma absent; veins ScP+R, MP and CuA separated at base, first fork of vein ScP+R near base; first fork of vein MP after first fork of vein CuA; clavus long, reaching near 4/5 of tegmina length; veins Pcu and A1 fused at basal half of clavus.

***Hind wings*** (Fig. 2E): translucent, with veins blackish; CuP-Pcu-A1 lobe weakly infuscate, with apical angle blackish; A2 lobe weakly infuscate and roundly protruding along postclaval margin.

***Venation***: main veins present; ScP+R, MP, and CuA running more or less parallel, slightly diverging towards posterior; Pcu curved around distal third of wing towards CuP but not reaching the latter; A2 complete, reaching margin.

***Legs*** (Figs 1A, B, 2F): pale yellowish brown, with dark brown marking. Metatibiotarsal formula: (2) 9 / 2 / 2.

***Abdomen*** (Fig. 1B): Pale yellowish brown.

***Terminalia*** ♂ (Fig. 3): pygofer (*Py* – Fig. 3A, B) ~1.6× as high as wide in caudal view, in lateral view with posterior margin deeply inclined posteriorly at upper 1/3 and deeply inclined forward and slightly sinuate at lower 2/3. Gonostyli (*G* – Fig. 3A–C) rather short in lateral view, with capitulum at apicodorsal angle, and with large laminate process behind capitulum; posteroventral angle rounded; capitulum (*ca* – Fig. 3A–E) laterally flattened, with posterior margin rounded in lateral view and 2 strong lateral spines derived from anterior portion, projecting lateroventrad, the dorsal one about twice as long as the ventral one. Aedeagus (Fig. 3F–I) upcurved, with pair of lateral pointed processes (*lpa*) not reaching apex, and 3 small terminal processes, middle one with small, triangular lamina projecting anterodorsad; dorsal process of periandrium (*dpp*) large, foliaceous, somewhat shovel-shaped, and concave in distal half, and with apical margin widely rounded; paired ventral processes of periandrium (*vpp*) surpassing ½ length of aedeagus, elongate, and with pointed apex curved lateroposterad; connective (*co*) strongly curved. Anal tube (*An* – Fig. 3 A–C) subcylindrical, with basal ventral bulge, ~1.9× as long as wide in dorsal view, with apical margin rounded in dorsal view, excavate in lateral view; epiproct short, located at distal third of anal tube.

**Female.** Similar to male.

##### Etymology.

The species epithet *bachmaensis* refers to Bach Ma National Park where the new species was discovered.

##### Biology.

The specimens were found sitting on leaves on the lower vegetation (Fig. 4A) in a subtropical evergreen forest (Fig. 4B) at the junction of the Northern Vietnam lowland rain forests, Southern Vietnam lowland rain forests, and Southern Annamites montane rain forests ecoregions, at rather low altitude (150–600 m).

##### Distribution.

Vietnam, Thua Tinh-Hue Province, Bach Ma National Park and Da Nang Province, Ba Na-Nui Chua Nature Reserve (Fig. 5).

#### ﻿ Connelicita haiphongensis

Taxon classificationAnimaliaHemipteraTropiduchidae

Wang & Zhang, 2015

0066FD3B-83CF-5110-9F0B-0B240E15A238

Figs 5 , 6

Connelicita haiphongensis Wang & Zhang, 2015 in Wang et al. 2015: 569, figs 2–21.

##### Materials examined.

Vietnam – **Hai Phong Province** • 1 ♂;Cat Ba National Park; 20°48'00"N, 107°00'20"E; 12–16 Jul. 2013; J. Constant & J. Bresseel leg.; I.G.: 32.454; RBINS – **Vinh Phuc Province** • 5 ♂♂, 5 ♀♀; Me Linh Biodiversity Station; 21°23'04"N, 105°42'44"E; 29–30 Jun. 2023; J. Constant & L. Semeraro leg.; RBINS • 2 ♂♂, 1 ♀; same locality as preceding; 29 Jun. 2023; alt. 30 m; by net; Hoai T.T. Nguyen leg.; VNMN – **Hoa Binh Province** • 1 ♂, 1 ♀;Ngoc Son-Ngo Luong Nature Reserve; 20°26'16"N, 105°20'15"E; 25–30 Jul. 2016; GTI Project; J. Constant & J. Bresseel leg.; RBINS – **Ninh Binh Province** • 1 ♀; Cuc Phuong National Park; 20°20'53"N, 105°35'52"E; 31 Jul.–3 Aug. 2016; GTI Project; J. Constant & J. Bresseel leg.; RBINS.

##### Note.

The species was previously only recorded from Cat Ba Island in Ha Long Bay (Wang et al. 2015). It is here recorded for the first time from the mainland, and its distribution is extended to the provinces of Vinh Phuc, Hoa Binh, and Ninh Binh (Fig. 5).

##### Biology.

**Figure 6.**
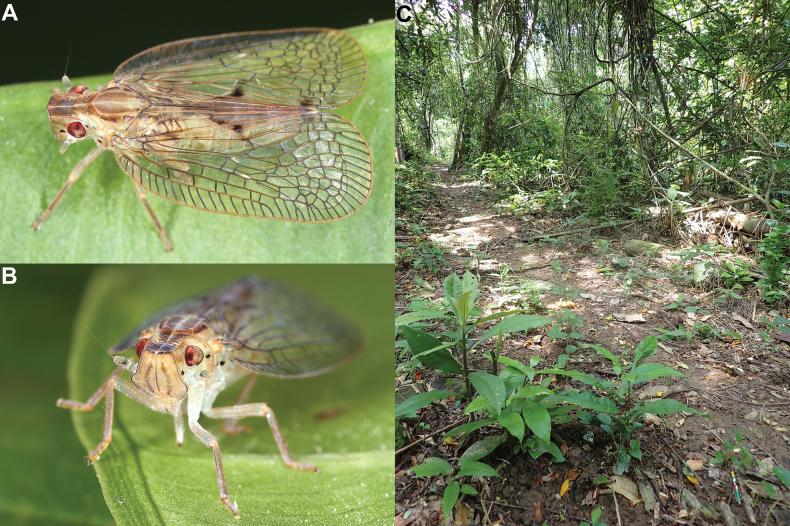
*Connelicitahaiphongensis* Wang & Zhang, 2015 **A, B** adult specimen on leave of unidentified plant, Vietnam, Cuc Phuong National Park, 4 July 2019 Gernot Kunz, with permission **A** laterodorsal view **B** anterodorsal view **C** habitat in Vietnam, Me Linh Biodiversity Station, 30 June 2023.

The specimens were found sitting on leaves on the lower vegetation (Fig. 6A, B) in subtropical evergreen forests (Fig. 6C) in the South China–Vietnam subtropical evergreen forests and Northern Indochina subtropical forests ecoregions.

#### ﻿ Connelicita phongdienensis

Taxon classificationAnimaliaHemipteraTropiduchidae

Constant & Pham sp. nov.

F762F99D-941B-58FD-AB41-B1771CF29320

https://zoobank.org/556D7EFE-52A9-4D76-869B-7C59FDDC754C

**Figure 7.**
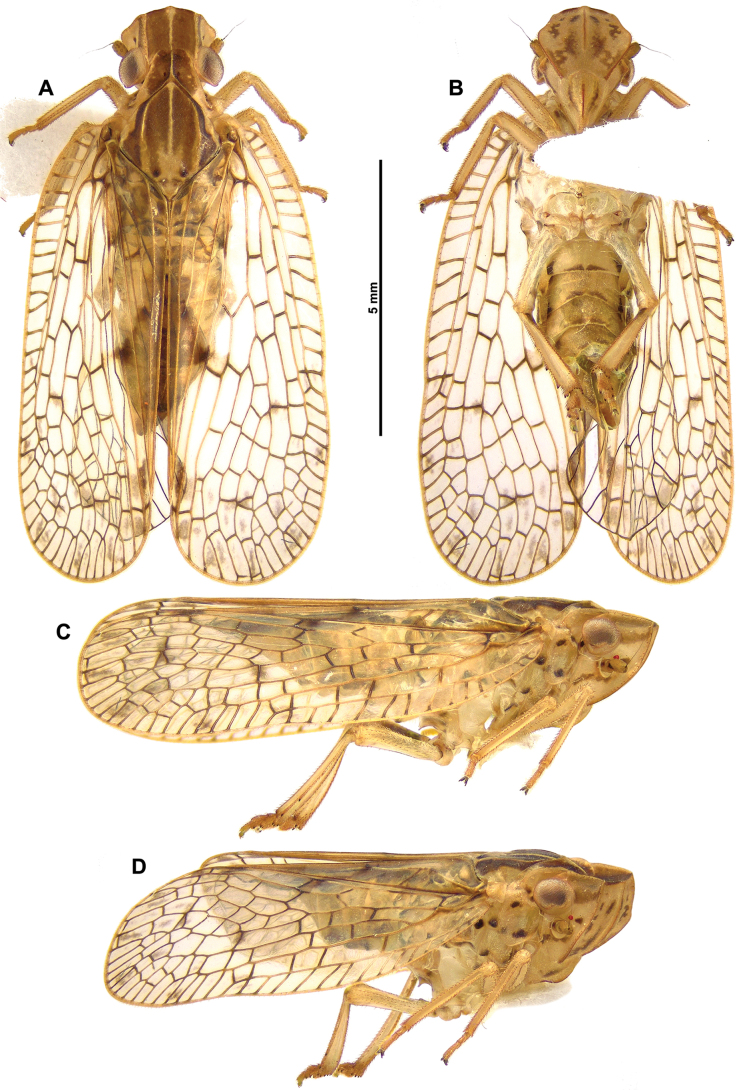
*Connelicitabachmaensis* sp. nov., holotype ♂ **A** habitus, dorsal view **B** habitus, ventral view **C** habitus, lateral view **D** habitus, anterolateral view.

**Figure 8.**
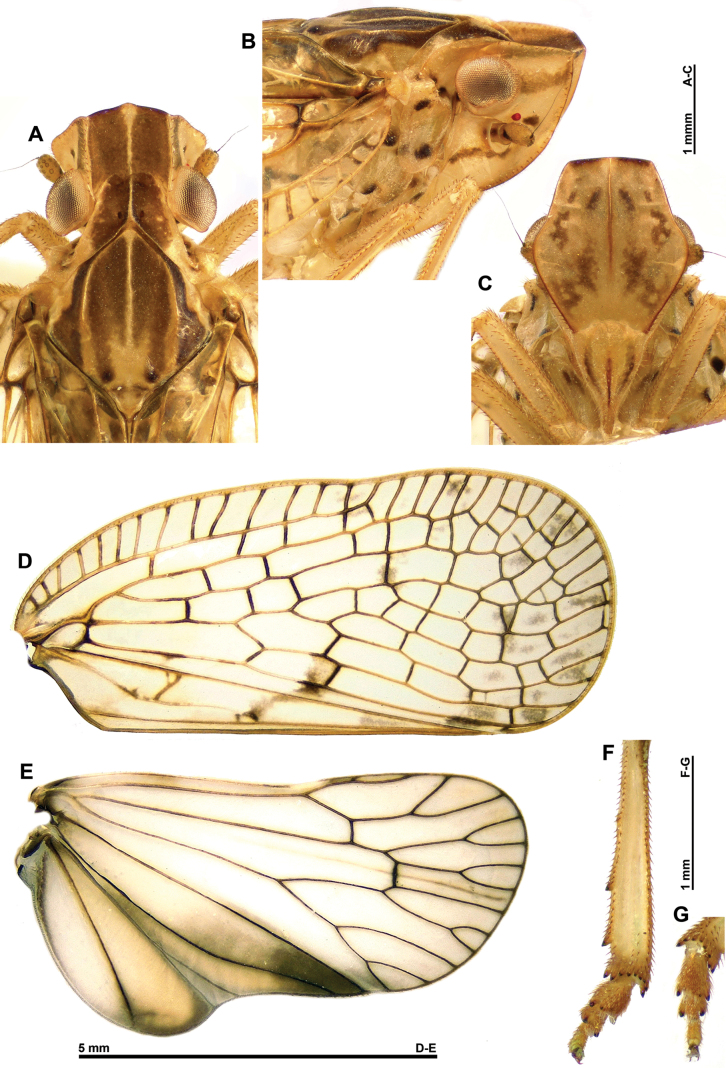
*Connelicitaphongdienensis* sp. nov., holotype ♂ **A–C** detail of head and thorax **A** dorsal view **B** lateral view **C** perpendicular view of frons **D** right tegmen **E** right hind wing **F** right metatibia and metatarsus, ventral view **G** left metatarsus, ventral view.

**Figure 9.**
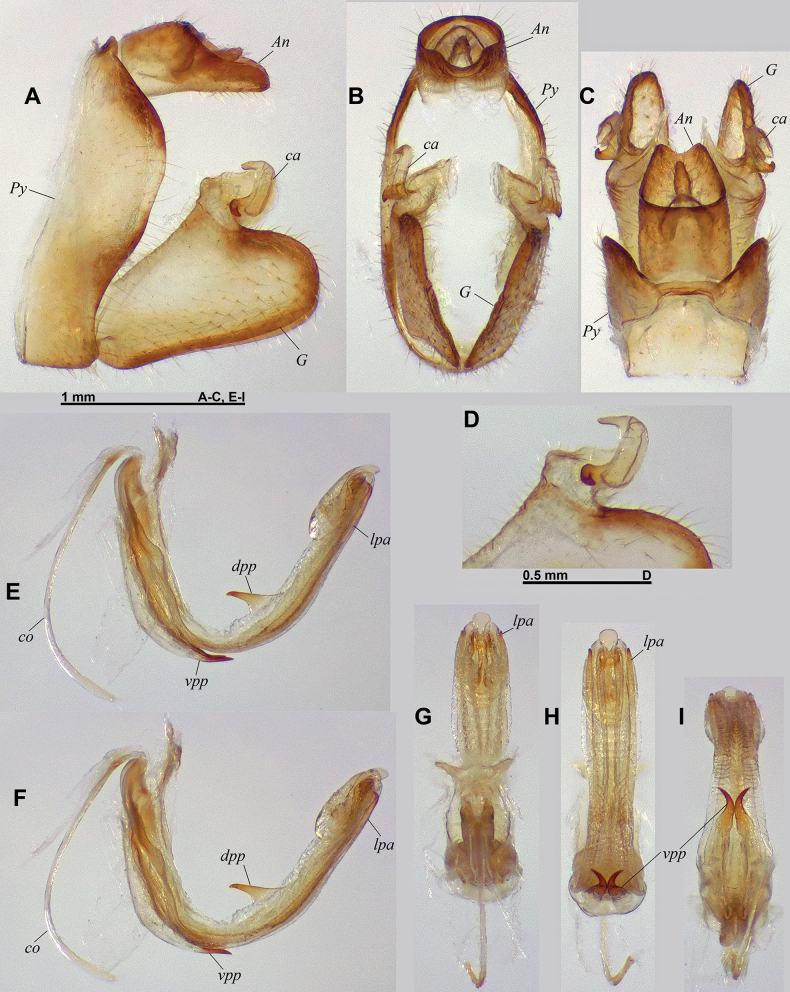
*Connelicitaphongdienensis* sp. nov., holotype ♂, terminalia **A–C** pygofer, gonostyli and anal tube **A** lateral view **B** caudal view **C** dorsal view **D–E** capitulum of left gonostylus **D** left laterodorsal view **E** right laterodorsal view **F–I** aedeagus, phallobase and connective **F** left lateral view **G** anterodorsal view **H** posteroventral view dorsal view **I** left lateroventral view.

**Figure 10.**
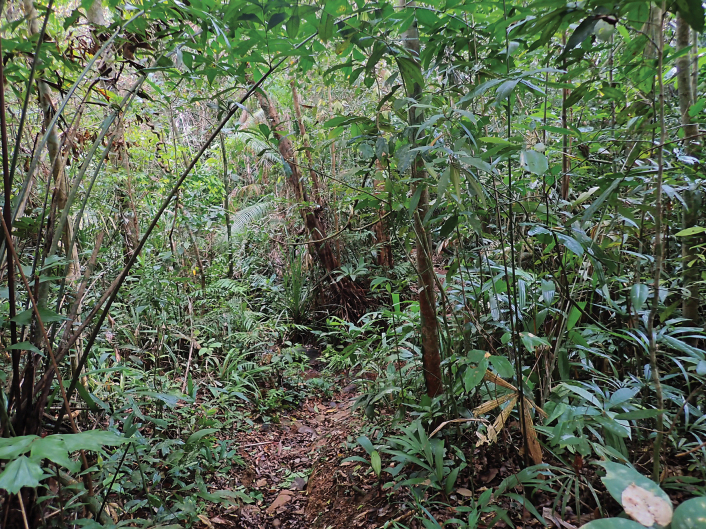
Habitat of *Connelicitaphongdienensis* sp. nov., Vietnam, Phong Dien, CCRR, 23 May 2023.

Figs 5 , 7 , 8 , 9 , 10

##### Type materials.

***Holotype*** ♂, Vietnam – **Thừa Thiên-Huế Province** • Phong Dien Nature Reserve, CCRR; 16°30'27"N, 107°16'05"E; 23 May 2023; alt. 350–400 m; J. Constant & L. Semeraro leg.; I.G.: 34.640; VNMN.

***Paratypes***, Vietnam •– **Thừa Thiên-Huế Province** • 1 ♂; same collection data as for holotype; RBINS • 1 ♀; Phong Dien Nature Reserve, CCRR; 16°30'27"N, 107°16'05"E; 23 May 2023; alt. 350–400 m; Trung T. Vu leg.; VNMN • 1 ♀; same collection data as for preceding; RBINS.

##### Diagnosis.

The species can be separated from the other species of *Connelicita* by the following characters of the male terminalia: anal tube in lateral view abruptly narrowing in distal half (Fig. 9A), posterior margin of pygofer rounded in lateral view (Fig. 9A), capitulum of gonostylus with a lateral spine in ventral portion, and a dorsal curved digitiform process (Fig. 9A, D), dorsal process of periandrium tooth-shaped, strong, and projecting dorsocephalad (*dpp* – Fig. 9E) and absence of a strong digitiform process directed dorsally at apex of aedeagus (Fig. 9E–G).

The closest species are *C.backyensis* and *C.haiphongensis*, from which *C.phongdienensis* Constant & Pham, sp. nov. can be separated by the dorsal digitiform process of the capitulum of the gonostylus, which is absent in both other species (compare with Wang et al. 2015: figs 12, 31).

##### Description.

***Measurements and ratios***: LT: ♂ (*n* = 2): 10.15–10.77 mm. LTg/BTg = 2.26; LW/BW = 1.89; LV/BV = 0.85; LF/BF = 1.04.

***Head*** (Fig. 8A–C): narrower than thorax and elongate, with ~2/3 of vertex length surpassing eyes and genae largely visible from above. Vertex brown, with median yellowish line, weakly concave, ~1.0× as long in mid-line as broad basally, concave on each side, with lateral margins subparallel, anterior margin roundly produced anteriorly in middle portion and posterior margin excavate. Frons yellowish brown, with irregular brown markings; convex in lateral view; smooth, with median carina reaching dorsal margin but not frontoclypeal suture. Genae yellowish, with brown marking between eye and anterior margin, brown line between antennal insertion and posterior margin, and a dark brown spot near red ocellus. Clypeus pale yellowish brown, with median carina and one curved line on each side, brown, triangular, with median carina in distal portion and with fronto-clypeal suture rounded. Labium yellowish, elongate, and narrow, reaching metacoxae, with apical segment elongate. Eyes globular (not emarginate) protruding laterally. Antennae yellowish brown, with longitudinal black line along underside of pedicel; scape ring-shaped and pedicel cylindrical, longer than broad.

***Thorax*** (Fig. 8A, B): Pronotum brown, with median carina, posterolateral angles and 3 or 4 small yellowish tubercles on sides of disc, yellowish; paranotal fields yellowish, with 3 black spots, one being behind eye. Mesonotum brown, with median and peridiscal carinae, anterior portion of lateral angles, scutellum, and area before latter yellowish; lateral fields darker and 2 blackish points at base of scutellum. Tegulae yellowish brown.

***Tegmina*** (Figs 7A–D, 8D): translucent, with brown spot near middle of vein CuP and vein CuA2, extending into clavus and irregular, greyish markings in cells in distal half of tegmen; costal and postclaval margins slightly diverging towards posterior; distal margin widely rounded.

Venation (Fig. 8D): costal area well developed, with numerous cross-veins delimitating elongate cells, pterostigma absent; veins ScP+R, MP, and CuA separated at base, first fork of vein ScP+R near base; first fork of vein MP after first fork of vein CuA; oblique, transverse cross-vein between MP and CuA1 in basal portion of latter; clavus long, reaching near 4/5 of tegmina length; veins Pcu and A1 fused at basal half of clavus.

***Hind wings*** (Fig. 8E): translucent, with veins blackish; CuP-Pcu-A1 lobe infuscate in distal portion, with apical angle largely blackish; A2 lobe weakly infuscate and roundly protruding along postclaval margin.

Venation: main veins present; ScP+R, MP, and CuA running more or less parallel, slightly diverging towards posterior; Pcu curved around distal third of wing towards CuP but not reaching the latter; A2 complete, reaching margin.

***Legs*** (Figs 7A, B, 8F–G): pale yellowish brown, with dark brown marking along dorsal portion of metafemora and basiventral portion of metatibiae. Metatibiotarsal formula: (2) 9 / 2 / 2.

***Abdomen*** (Fig. 7B): pale yellowish brown, with narrow brown line interrupted in middle, along posterior margin of sternites.

***Terminalia*** ♂ (Fig. 9): pygofer (*Py* – Fig. 9A, B) ~1.9× as high as wide in caudal view, in lateral view with posterior margin deeply inclined posteriorly at upper 1/3 then broadly rounded, and sinuate at lower 2/3. Gonostyli (*G* – Fig. 9A–C) rather elongate in lateral view, with capitulum at about 2/3 of gonostylus length, and with large laminate process projecting medially behind capitulum; posterior lobe rounded; capitulum (*ca* – Fig. 9A–D) laterally flattened, with posterior margin rounded in lateral view and bearing a complex lateral process, with upper digitiform process curved cephalodorsad and sinuate ventral spine directed posteroventrad. Aedeagus (Fig. 9E–I) strongly upcurved at mid-length, with pair of slender lateral pointed processes (*lpa*) not reaching apex of aedeagus, and with apical point directed anterodorsad; 3 small terminal processes, middle one shortly projecting posterad; dorsal process of periandrium (*dpp*) tooth-shaped, strong and projecting dorsocephalad, placed slightly after mid-length of aedeagus; paired ventral processes of periandrium (*vpp*) not reaching ½ length of aedeagus, elongate, sinuate in distal portion, and with pointed apex directed lateroposterad; connective (*co*) moderately curved. Anal tube (*An* – Fig. 9A–C) ~1.26× as long in median line, as wide, in dorsal view; subcylindrical in proximal half, then with dorsal margin excavate and sinuate in lateral view; in dorsal view, lateral margins subparallel in proximal half, then regularly converging in distal half; posterior margin deeply concave in dorsal view, with basal ventral bulge; epiproct rather large, located at ½ length of anal tube.

**Female.** Similar to male.

##### Etymology.

The species epithet *phongdienensis* refers to Phong Dien District, the locality where the new species was discovered, at the Centre for Conservation of Vietnam Natural Resources and Rescue of Animals and plants.

##### Biology.

The specimens were found sitting on leaves on the lower vegetation in a subtropical evergreen forest (Fig. 10) in the Northern Vietnam lowland rain forests ecoregion at rather low altitude (150–600 m).

##### Distribution.

Vietnam, Thua Tinh-Hue Province, Phong Dien District, CCRR (Fig. 5).

## ﻿Discussion

The present work adds two species of *Connelicita* to the fauna of Vietnam, leading to a total of four species for the country, while one additional species is known from China. The new records also greatly extend the distribution of the genus to the south but leaves a gap of more than 500 km without any record of *Connelicita* in the northern half of Central Vietnam where new species will certainly be discovered in the future. Although the species are easily identified from male terminalia characters, their external morphology is extremely similar, and the species should not be identified based only on photographs. Citizen-science platforms like iNaturalist should refrain from their members identifying such insects to species level, unless good photographs of the genitalia of the corresponding specimen are provided to support the identification.

In Phong Dien District, VNMN is conducting an ambitious project of forest restauration at the Centre for Conservation of Vietnam Natural Resources and Rescue of Animals and plants, not far from the forest where *C.phongdienensis* Constant & Pham sp. nov. was discovered. The return of such planthopper species in this area in the future would be a great indicator of a successful project.

## Supplementary Material

XML Treatment for Connelicita

XML Treatment for Connelicita bachmaensis

XML Treatment for Connelicita haiphongensis

XML Treatment for Connelicita phongdienensis

## ﻿Additional information

### Author ORCIDs

Jérôme Constant https://orcid.org/0000-0003-0254-0863

Thai-Hong Pham https://orcid.org/0000-0002-4763-3679

Cuong Viet Canh Le https://orcid.org/0000-0002-1430-6305

Trung Thanh Vu https://orcid.org/0009-0002-8987-1672

Hoai Thu Thi Nguyen https://orcid.org/0009-0004-9130-2940

### Data availability

All of the data that support the findings of this study are available in the main text.

## References

[B1] Bourgoin T (2023) FLOW (Fulgoromorpha Lists on the Web): a world knowledge base dedicated to Fulgoromorpha. V.8, updated [i.2023]. http://hemiptera-databases.org/flow/ [Accessed on: 2023-09-12]

[B2] Bourgoin T Huang J (1990) Morphologie comparée des genitalia mâles des Trypetimorphini & remarques phylogénétiques (Hemiptera: Fulgoromorpha: Tropiduchidae). Annales de la Société entomologique de France. Nouvelle Série 26(4): 555–564. 10.1080/21686351.1990.12277614

[B3] Bourgoin T Wang RR Asche M Hoch H Soulier-Perkins A Stroiński A Yap S Szwedo J (2015) From micropterism to hyperpterism: recognition strategy and standardized homology-driven terminology of the fore wing venation patterns in planthoppers (Hemiptera: Fulgoromorpha). Zoomorphology 134(1): 63–77. 10.1007/s00435-014-0243-6 25705075 PMC4326643

[B4] Constant J (2004) Révision des Eurybrachidae (I). Le genre *Amychodes* Karsch, 1895 (Homoptera: Fulgoromorpha: Eurybrachidae). Bulletin de l’Institut royal des Sciences naturelles de Belgique 74: 11–28.

[B5] O’Brien LB Wilson SW (1985) Planthoppers systematics and external morphology. In: Nault LR Rodriguez JG (Eds) The Leafhoppers and Planthoppers.John Wiley & Sons, New York, 61–102.

[B6] Shorthouse DP (2010) SimpleMappr, an online tool to produce publication-quality point maps. http://www.simplemappr.net [Accessed 28 September 2023]

[B7] Wang M Stroiński A Bourgoin T Zhang Y (2015) A new Asian genus of the tribe Elicini (Hemiptera: Fulgoromorpha: Tropiduchidae) with two new species from Vietnam. Zootaxa 4018(4): 563–572. 10.11646/zootaxa.4018.4.5 26624056

